# Supplementary material for: Use of a Silkworm (Bombyx mori) Larvae By-Product for the Treatment of Atopic Dermatitis: Inhibition of NF-κB Nuclear Translocation and MAPK Signaling
Source: Nutrients. 2023 Apr 5;15(7):1775. doi: 10.3390/nu15071775 (PMC10097122; doi:10.3390/nu15071775)
Supplement: Supplementary file 1 [file nutrients-15-01775-s001.zip › nutrients-2296424-supplementary.pdf]

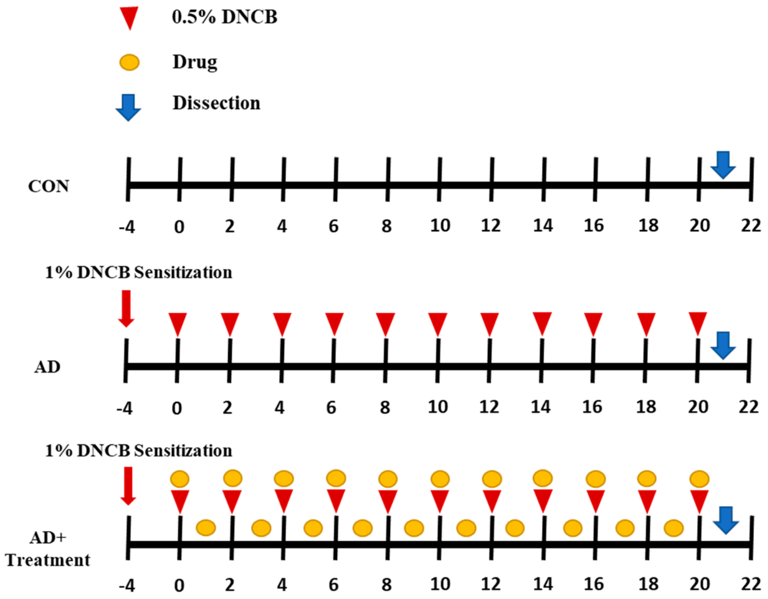

Supplementary Figure S1 Experimental design for DNCB-induced BALB/c mice. Four days before topical application of SLPY, 1% DNCB applied to evoke AD-like skin lesions. After 4 days of 1% DNCB sensitization, mice were challenged with 0.5% DNCB for two days interval for 3 weeks. Two concentration levels (50, 100 mg/kg) of SLPY and dermatop (0.25% (w/w) topically applied daily to treat AD-like skin lesion and compare against the control and AD groups.

Supplementary Table S1 Primer sequence of the respective gene used in qRT-PCR analysis

Gene	Primer	Sequence
**GAPDH**	Forward	GCA CAG TCA AGG CCG AGA AT
	Reverse	GCC TTC TCC ATG GTG GTG AA
**TNF-α**	Forward	AAG CCT GTA GCC CAC GTC GT
	Reverse	GGC ACC ACT AGT TGG TTG TC
**IL-4**	Forward	ACA GGA GAA GGG ACG CCA T
	Reverse	GAA GCC GTA CAG ACG AGC TC
**IL-6**	Forward	CCG GAG AGG AGA CTT CAC AG
	Reverse	GGA AAT TGG GGT AGG AAG GA
**IL-10**	Forward	TCA GCT GTG TCT GGG CCA CT
	Reverse	TTA TGA GTA GGG ACA GGA AG
**IL-13**	Forward	GCA ACA TCA ACA GGA CCA GA
	Reverse	GTC AGG GAA TCC AGG GCT AC
**IL-17**	Forward	TCC CCT CTG TCA TCT GGG AA
	Reverse	CTC GAC CCT GAA AGT GAA GG
